# MR-Guided Focused Ultrasound Central Lateral Thalamotomy for Trigeminal Neuralgia. Single Center Experience

**DOI:** 10.3389/fneur.2020.00271

**Published:** 2020-04-17

**Authors:** Marc N. Gallay, David Moser, Daniel Jeanmonod

**Affiliations:** SoniModul, Center for Ultrasound Functional Neurosurgery, Solothurn, Switzerland

**Keywords:** trigeminal neuralgia, trigeminal pain, MR-guided high intensity focused ultrasound, central lateral thalamotomy, stereotactic functional neurosurgery

## Abstract

**Background:** Trigeminal neuralgia (TN) is a recognized pain condition the treatment of which can be very challenging. Various surgical interventions can be applied in cases of therapy-resistance to drug treatments. The central lateral thalamotomy (CLT) against neurogenic (or neuropathic) pain is based on multiarchitectonic histological as well as physiopathological studies, and integrates the nucleus in a large thalamocortical (TC) and corticocortical network responsible for the sensory, cognitive and affective/emotional components of pain. The advent of the magnetic resonance imaging guided high intensity focused ultrasound (MRgFUS) brought a strong reduction in morbidity and increase in accuracy compared to penetration techniques.

**Objective:** This study was aimed at analyzing the outcome of bilateral MRgFUS CLT for chronic therapy-resistant trigeminal pain, all performed in one single center.

**Methods:** Patients were categorized in Classical, Idiopathic and Secondary TN. By definition, paroxysms lasted for seconds up to 2 min. All patients were screened for trigeminal neurovascular conflict. In case of classical TN, microvascular decompression was proposed. Therapy-resistance and thus indication for MRgFUS CLT was based on the lack of efficacy and/or side effects of antiepileptic and antidepressant drugs. Good outcome was defined by a pain relief ≥50%.

**Results:** Eight patients suffering from chronic therapy-resistant trigeminal neuralgia were treated. All suffered from pain with paroxysmal character. Six patients reported additionally continuous pain. Mean follow-up was 53 months (range: 12–92, median: 60 months). The mean pain relief assessed by patients was 51% (median: 58%, range: 0–90%) at 3 months, 71% (median: 65%, range: 40–100%) at 1 year and 78% (median: 75%, range: 50–100%) at their longest follow-up. This represents 63% good outcomes at 3 months, 88% at 1 year and 100% at last follow-up. Frequency of the mean pain paroxysms decreased from 84 per day preoperative to 3.9 at 1 year postoperatively. There were no serious adverse events in this series.

**Conclusion:** Our study provides preliminary support for the safety and efficacy of MRgFUS CLT, a histologically and pathophysiologically based medial thalamotomy against chronic therapy-resistant trigeminal neuralgia.

## Introduction

At the beginning of the 20th century, Head and Holmes postulated the presence of an “essential medial thalamic center,” anatomically located medially to a pain-generating lesion in the thalamic ventral posterior complex (VP) and responsible for the pathogenesis of central pain (1). Sano proposed the generation of abnormal impulses in VP and their amplification in a reverberating circuit between lateral and medial thalamic nuclei (2). Experimental and clinical data reported by Cesaro et al. (3) and Pierre et al. (4) supported an imbalanced interaction between medial and lateral thalamic areas, with a postulated disinhibition of the medial thalamus. The medial thalamotomy was one of the first stereotactic interventions performed on the human brain in the early 1950s. Unlike other lesional surgeries, medial thalamotomies against neurogenic (or neuropathic, or de-afferentation) pain have been recognized as interventions with a low complication rate and without the risk for developing iatrogenic pain manifestations or somatosensory deficits. They have been shown to provide pain relief for all body locations, and bilateral medial thalamotomies were shown to be more efficient than unilateral contralateral ones (5–7). This is in concordance with the fact that thalamic low threshold calcium spike bursts (6, 8) were found bilaterally and quantitative electroencephalographic (EEG) recordings showed evidence of bilateral physiopathology (see below). Although cases of total and stable pain relief have been published, recurrence of initial pain was frequent (2, 7, 9–16). These first reports led us from the late 1980s onward to re-investigate the medial thalamus and finally establish the posterior part of the Central Lateral nucleus (CLp) as target in chronic therapy-resistant neurogenic pain (5, 6, 8, 17–21), The central lateral thalamotomy (CLT) as a surgical intervention against neurogenic pain is based on multiarchitectonic histological studies and integrated in a large thalamocortical (TC) network responsible for the sensory, cognitive and affective/emotional components of pain. The CLp is in a position to transfer nociceptive information's conveyed through the spinothalamic and spinoreticulothalamic pathways to relatively large domains of cortex, including areas involved in nociception, mainly SII, insula and anterior cingulate cortex. In addition, single unit recordings of CLp thalamic cells (5, 6, 8, 18) and quantitative EEG and MEG analyses (22–24) have demonstrated TC overactivities located on cortical pain areas, constituting the final product of a TC process named thalamocortical dysrhythmia. This process is based on the de-afferentation of thalamic cells, which causes an increase of EEG low and high frequency activities at the source of pain perception. These microphysiological and quantitative EEG/MEG studies have shown the same pathophysiology for all neurogenic pain syndromes, whatever their location in the body, and thus including the trigeminal location. The absence of somatosensory deficits in most of the classical TN patients is likely due to the great compensatory capacities of the peripheral sensory trigeminal system and of the thalamocortical network, in addition to limitations of sensitivity of the physical examination.

The results obtained years ago in the medial thalamus by a few neurosurgical groups tend to support the primacy and possible exclusivity of CLp as a regulatory medial thalamic target: Sano (2), as an exception in his time, focused his efforts on the posterior part of the medial thalamus using a posterior approach, thus approaching more than anyone else the CLp, which was not or only partly reached by others. Hitchcock and Teixeira (7) as well as Young and col. (25) placed relatively large lesions in the posterior part of centrum medianum (CM)/Parafascicular nucleus (Pf), probably involving parts of the CLp. Urgosik and Liscak recently reported an overall pain relief success rate in 43% of their patients targeting the medial thalamus (CM/Pf complex) with the gamma knife (26). Those results were recently replicated by another group (27).

Since the first clinical experience with the MRgFUS (28) and a series with 1 year follow-ups against neurogenic pain (19), safety and accuracy data on this technique have been published several times (29–31).

This case series analyze the clinical results of consecutive MRgFUS treatments performed for chronic therapy-resistant trigeminal neuralgia with a mean follow-up of 4 years. This report reflects our current practice of treating chronic therapy-resistant neurogenic pain regardless of which body part is involved.

## Methods

All patients treated with this protocol signed an informed consent form after having been fully informed about the treatment, its results and risks. No additional ethical approval was sought because MRgFUS CLT has been approved by the Federal Office of Public Health (FOPH) of Switzerland and is covered by swiss social insurances.

Patients were categorized according to Cruccu et al. (32) in Classical TN, Idiopathic TN and Secondary TN. Classical TN is defined as a specific category of TN in which MR demonstrates vascular compression of the trigeminal nerve root, Idiopathic TN occurs without apparent cause and secondary TN is the consequence of a major neurological disease (32). Outcome measures followed the criteria proposed by Zakrzewska and Lopez (33). By definition, paroxysms lasted from seconds up to 2 min. All patients were screened for trigeminal neurovascular conflict. In the case of such a conflict, microvascular decompression was proposed. Therapy-resistance and thus indication for MRgFUS CLT was based on the lack of efficacy and/or side effects of antiepileptic and antidepressant drugs during at least a year. Diagnosis was always ascertained by at least one neurologist. All Swiss patients operated between 2015 and 2017 were included in the Swiss registry for the incisionless MRgFUS therapy in functional neurosurgery and were seen postoperatively by an independent neurologist. Antiaggregant therapy was stopped 10 days before the intervention. Normal coagulation and blood pressure were checked for all patients prior to surgery.

### Surgical Procedure and Target Determination

**Figure 1:**
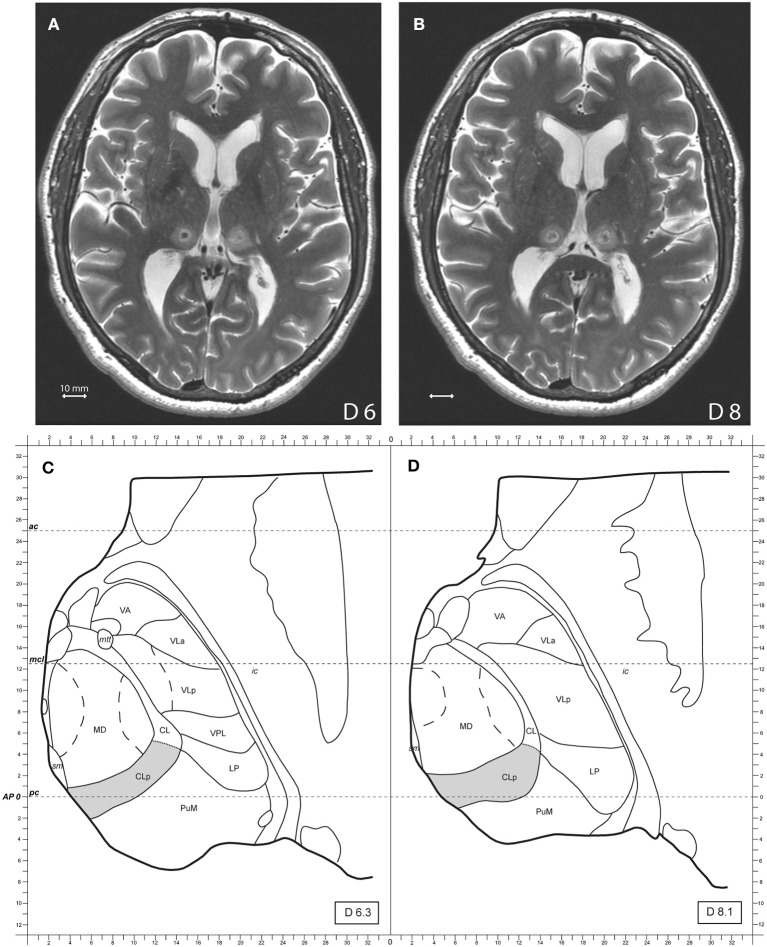
**(A,B)** Show axial MR T2 images two days after the treatment, 6 and 8 mm dorsal to the intercommissural plane of a bilateral MRgFUS CLT. **(C,D)** Show modified atlas maps of the Morel's Atlas 6.3 and 8.1 mm dorsal to the intercommissural plane with the posterior Central Lateral nucleus (CLp) in gray. Mammillothalamic tract (mtt), ventral anterior nucleus (VA), ventral lateral anterior nucleus (VLa), ventral lateral posterior nucleus (VLp), ventral posterior lateral nucleus (VPL), lateral posterior nucleus (LP), medial pulvinar (PuM), mediodorsal nucleus (MD), internal capsule (ic), posterior commissure (pc), anterior commissure (ac).

The surgical procedure using the MRgFUS to perform CLT (19, 28), target reconstruction and accuracy determination (29–31) were described in prior publications. CLT was planned on maps of the Morel's Atlas of the human thalamus and Basal Ganglia (21) and modified according to individual anatomy as seen on the preoperative MR high resolution images cut in stereotactic planes. Target determination and coverage of the CLT target evolved over the years of clinical experience with at first placement where CLp output fibers converge, i.e., one sonication spot 6 mm dorsal to the intercommissural plane and 8 mm from the medial thalamic border. Our present and latest targeting strategy has as a goal to optimize CLT target coverage and consists of a set of 4 target sub-units placed at 6 mm (2 sub-units) and 8–9 mm (2 sub-units) dorsal to the intercommissural plane. The anteroposterior position of the sub-units is determined based on visualization on preoperative MR T2 axial images of the junction between the medial dorsal nucleus (MD) and medial pulvinar (PuM) corresponding to the position of the CLp, centered in our experience between 3 mm anterior and 1 mm posterior to the posterior commissure. In the mediolateral (ML) dimension, 2 sub-units are placed to cover the ML extent of the CLp, i.e., from medial thalamic border to 10 mm laterally, e.g., 5 and 8 mm laterally for ML position of the sub-unit centers. Figure 1 shows a bilateral MRgFUS CLT.

Ten mg domperidone (Motilium lingual®) were given prior starting sonications. The last patient of this series, received in accordance to our actual routine operation protocol 20 mg intravenous methylprednisolone in the hour following the end of the operation, 20 mg after 12 h and dexamethasone 2 mg three times daily for 3–4 days in order to control/limit the perifocal edema of the lesion. Control MR was performed 2 days postoperatively. Accuracy determination and target reconstruction were performed according to Moser et al. (30, 31).

### Follow-Up

Detailed pain assessments with a full neurological examination including assessment of esthesia and algesia were performed preoperatively and postoperatively after two days, 3 months and 1 year. Later follow-up assessments were mostly performed through e-mail and phone conversations. Pre- and postoperative assessments included the items of the McGill Pain Questionnaire. Pain intensity was noted on a visual analog scale (VAS) for the least, the worst and mean pain intensities on a scale between 0 and 100. Patients were asked for a percentage value of postoperative pain relief as compared with their preoperative state. Mini-mental test and later Montreal Cognitive Assessments were performed preoperatively and after 2 days and 1 year follow-up. Good outcome was defined by a pain relief ≥50%. A recurrence was defined as initial good outcome (pain relief ≥ 50%) and later decrease of pain relief <50% and/or recurrence of pain attacks.

### Statistics

Statistical analysis of quantitative scores compared with baseline was carried out by repeated ANOVA measures and multiple comparisons were applied using a *post hoc* analysis with Bonferroni-Holm testing (Daniel's XL toolbox; https://www.xltoolbox.net/).

## Results

Patient's characteristics are summarized in Table 1. Mean symptoms duration was 17 ± 12 years (range 4–37). Mean age at treatment was 62 ± 12 years (46–79). Three patients were female. Mean follow-up was 53 ± 35 months (12–92). Median follow-up was 60 months. No patient was lost to follow-up.

**Table 1:** Patients characteristics.

**Patient no**.	**Pain duration (yrs)**	**Side**	**Pain location**	**Etiology**	**Targets**	**Previous interventions**	**Primary headache history**	**Last follow-up (months)**
1	4	Right	V1,V2,V3	I	CLT bilat	–	–	90
2	37	Left	V2,V3	I	CLT bilat	Thermocoagulation	Migraine	92
3	12	Right	V1,V2,V3	C	CLT bilat	–	–	84
4	21	Left	V1,V2	S (Tumor)	CLT R*+ CMT R	Bilateral RF CLT	Tension-type headache	62
5	30	Right	V2,V3	I	CLT bilat	Thermocoagulation	–	58
6	6	Left	V1,V2,V3	S (MS)	CLT R^†^	Bilateral MRgFUS CLT	–	14
7	20	Right	V1,V2,V3	C	CLT bilat	MDV, Glycerol rhizotomy, 2 Thermocoagulations, GKS	–	15
8	4	Right	V3	C	CLT bilat	–		12
Mean (SD)	17 (12)							53 (35)
Median	16							60

*complement of radiofrequency ablation of the Central Lateral posterior nucleus (RF CLT), †complement of right CLT. C: classical. GKS, Gamma knife radiosurgery; I, idiopathic; MS, Multiple sclerosis; MVD, Microvascular decompression; S, secondary.

Eight consecutive trigeminal neuralgia patients treated between 06/2011 and 11/2017 were analyzed here. All patients suffered from pain with paroxysmal character. 6 patients reported additionally continuous pain. Three patients were classified as Idiopathic TN, 3 as Classical TN and 2 as Secondary TN. Secondary causes for TN were multiple sclerosis (1) and 1 trigeminal schwannoma operated 20 years prior to MRgFUS intervention. There has been no sign of recurrence The patient with multiple sclerosis did not present MR signs of active demyelination, i.e., plaques accounting for a new potential source of pain in addition to the known causal brainstem plaque. All patients showed at least mild somatosensory deficits at detailed clinical examination. Nine surgical interventions for pain were performed in 5 patients previously (4 trigeminal thermocoagulation, 1 microvascular decompression, 1 glycerol injection, 1 gamma knife irradiation of the root of the trigeminal nerve, and 1 bilateral radiofrequency CLT and 1 bilateral CLT with MRgFUS). Two patients with classical TN refused microvascular decompression (MVD) prior to this study time.

All patients had unilateral pain syndromes, 5 of them on the right side. Distribution of the pain in the trigeminal territories is given in Table 1.

### Surgery

Bilateral CLT in one session was performed in 6 patients. In Patient 4 previously treated with bilateral CLT RF, unilateral CLT complement as well as 1 centrum medianum (CM) target were performed. Patient 6 received a complementation on the right side of his bilateral MRgFUS CLT performed 14 months previously. The complement of the CLT target was offered because of symptom recurrence due to partial target coverage.

Average number of sonications was 15 ± 8 (5–31) and their duration was between 20 and 31 s. The average power of final sonications was 1020 ± 236 [W] (650–1300). Final temperatures were between 54 and 58°C. Mean lesion volume measured on MR T2 axial and sagittal images 2 days after treatment was 153 ± 85 mm^3^ (51–247 mm^3^). All patients were discharged after one night hospital stay.

### Pain Relief

**Figure 2:**
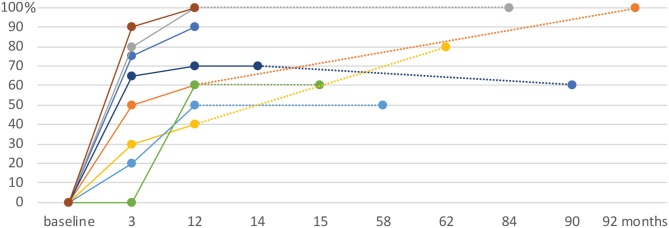
Pain relief in % as rated by the patients over time (months), from baseline to the last follow-up. Intervals between consultations of more than 12 months are connected with dotted lines.

The mean pain relief assessed by patients was 51% (median: 58%, range: 0–90%) at 3 months, 71% (median: 65%, range: 40–100%) at 1 year and 78% (median: 75%, range: 50–100%) at their longest follow-up (see Table 2 and Figure 2). This represents 63% good outcomes at 3 months, 88% at 1 year and 100% at last follow-up. As defined above, no patients had a recurrence during the study period. Patient 6, who had a recurrence after a previous bilateral MRgFUS CLT enjoyed a 60% pain relief 1 year after right-sided CLT target complementation. Between 3 months and 12 months, 2 patients went from an insufficient to a good pain outcome. One patient had insufficient pain relief (40%) at 1 year, but reached 80% at last follow-up (62 months).

**Table 2:** Summary of pain reliefs, baseline and postoperative pain intensities.

**Patient no**.	**Pain relief (%) at 3 months**	**Pain relief (%) at 1 year**	**Pain relief (%) last follow-up**	**Somatosensory deficits preoperatively**	**Sensory improvements at 1 year**	**baseline cont. pain min-max VAS**	**baseline cont. pain mean VAS**	**baseline pain paroxysms min-max VAS**	**baseline pain paroxysms mean VAS**	**3 months cont. pain min-max VAS**	**3 months cont. pain mean VAS**	**3 months pain paroxysms min-max**	**3 months pain paroxysms mean VAS**	**1 year cont. pain min-max VAS**	**1 year cont. pain mean VAS**	**1 year pain paroxysms min-max VAS**	**1 year pain paroxysms mean VAS**	**last follow-up cont. pain min-max VAS**	**last follow-up cont. pain mean VAS**	**last follow-up pain paroxysms min-max VAS**	**last follow-up pain paroxysms mean VAS**
1	75	90	60	+	+	27–55	41	62–99	81	0–60	30	0	0	0–20	10	0	0	0–55	28	0	0
2	50	60	100	+	0	44–91	68	52–90	71	10–60	35	0	0	0–50	25	0	0	0	0	0	0
3	80	100	100	+	+	no	no	35–100	68	–	–	5–20	13	–	–	0	0	–	–	0	0
4	30	40	80	+	0	38–65	52	43–97	70	0	0	84	84	0	0	84–84	84	0	0	84	84
5	20	50	50	+	+	16–86	52	26	26	8–78	43	18	18	0–74	37	34–34	34	21–80	67	44	44
6	0	60	60	+	0	21–44	33	58–82	70	0	0	82	82	0	0	24–58	41	0	0	24–58	41
7	65	70	70	+	+	12–100	56	95–100	98	21–45	28	25–65	45	0	0	15–60	38	0	0	15–60	38
8	90	100	100	+	+	no	no	80	80	–	–	12	12	–	–	10	10	–	–	10	10
Mean	51	71	78				50		70		23		32		12		26		16		27
Median	58	65	75																		
											*p* = 0.01		*p* = 0.02		*p* = 0.0008*		*p* = 0.003*		*p* = 0.02		*p* = 0.005*

*P-values given after Bonferroni–Holm correction for multiple testing with baseline*.

* *values which reached statistical significance after correction*.

At last follow-up, pain paroxysms were still present in 5 patients (63%) but their mean intensity was 27 ± 30/100 compared to 70 ± 20 preoperatively on VAS. Of the 6 patients reporting continuous pain preoperatively, 2 still reported continuous pain at last follow-up. Their mean continuous pain level was 16 ± 27/100 at last follow-up, compared with 50 ± 12/100 preoperatively. At last follow-up, statistical significance was reached for pain paroxysms but not for continuous pain. Pain qualities of both (continuous and paroxysmal) pain components as well as frequency of spontaneous pain paroxysms are detailed in Table 3. Frequency of spontaneous pain paroxysms decreased from 84 (2–240) daily preoperatively, to 4.0 (0–2) at 3 months and 3.9 (0–23) 1 year postoperatively. Sensory improvements (reduction of esthesia and/or algesia deficits) were documented during postoperative clinical neurological examinations in 5 patients.

**Table 3:** Pain qualities at baseline and follow-up examinations and Frequency of pain paroxysms.

**Patient no**.	**Pain qualities preoperative**	**Pain qualities at 3 months FU**	**Pain qualities at 1 year FU**	**preoperative frequency of spontaneous pain paroxysms [d^**−1**^]**	**3 months FU frequency of spontaneous pain paroxysms [d^**−1**^]**	**1 year FU frequency of spontaneous pain paroxysms [d^**−1**^]**	**last control FU frequency of spontaneous pain paroxysms [d^**−1**^]**
1	B, P, E, L	T, B	B	240	0	0	0
2	St, P, B, E, L, C, T	P, B, C	B, S	8	0	0	0
3	E, L, C, T	0	0	*	*	0	0
4	S, St, B, C	Cut, C	T, B, S	3.5	2.0	0.5	0.1
5	P, S, B, E, T	P, B, L	P, A, L	2	0.03	0.05	0.02
6	P, E, C, T	St	E, A, T	100	*	*	*
7	E, B	E, P, B	B, P	150	–	23	23
8	P, A	P	P	*	*	*	*
Mean (SD)				84 (98)		4.0 (9.3)	3.9 (9.4)

Pins and needles (P), tearing (T), stinging (S), aching (A), burning (B), stabbing (St), compression (C), electricity (E), lightning (L), cutting (Cut), ^*^provoked attacks only. Follow-up (FU).

### Secondary Outcome Measures

Mean Anxiety and Depression Scale (HADS) scoring was 14.4 ± 5.8 (5–21) preoperatively, 8.5 ± 3.7 (2–14) at 3 months (*p* = 0.03) and 7.6 ± 4.1 (2–16) (*n* = 8, *p* = 0.017) at 1 year follow-up. They were no cognitive changes, as assessed with MMST (*n* = 3) or MoCA (*n* = 5). Mean MMST scores were 29.3 ± 0.6 preoperatively, 29.7 ± 0.6 at 2 days, 3 months and 1 year follow-up. Mean MoCA scores were 26.8 ± 4.1 (20–30) preoperatively, 28.0 ± 2.5 at 2 days (24–30) and 28.6 ± 2.1 (25–30) (*p* = 0.28) at 1 year follow-up.

### Morbidity

There were no serious adverse events in this series. Sonications were painful for a few seconds in 2 patients. No patients reported lasting headache >6 h after the procedure. There were 3 mild side effects, one postoperative frontal scalp swelling which resolved within a week and 2 mild cases of transient vertigo. There were no new somatosensory deficits, bleeding, infection or mortality in this series.

### Drugs

The drug intake of all patients was detailed in Table 4. Antiepileptic drug intake could be stopped in 2 patients and reduced in 2.

**Table 4:** Drug intakes.

**No**.	**tried drugs, already stopped**	**preoperative drug intake**	**3 months drug intake**	**1 year drug intake**	**drug intake at last follow-up**
1	Pregabaline various opiates	Carbamazepine 1,200 mg Anafranil 75 mg Tramadol and Buprenorphine-Patch	Carbamazepine 800 mg Anafranil 75 mg	Carbamazepine 800 mg	Carbamazepine 1,200 mg
2	Carbamazepine Opiates	Tramadol 200mg Ibuprofen 1800mg Pregabaline 100 mg Rivotril 1 mg	Pregabaline 100 mg Venlafaxine 150 mg Rivotril 0,5 mg	Pregabaline 100 mg Venlafaxine 150 mg	0
3	Amitriptyline	Carbamazepine 800mg Gabapentine 600 mg	Carbamazepine 600 mg	Carbamazepine 200mg	0
4	Pregabaline	Diclofenac	0	0	0
5	Carbamazepine, Pregabaline Gabapentine, Tramal, Amitriptyline, Naproxen, Paracetamol, Tizanidin	Durogesic-Patch 25 und 12 μg Targin i.R. Venlaflaxine 75 mg Trimipramine 25 mg	Targin 5 mg Trimipramine 25 mg	Targin 5 mg Trimipramine 25 mg	Oxynorm 1-0-1* Trimipramine 25 mg
6	–	Carbamazepine CR 1000mg, Pregabaline 200mg, Clomipramine 50 mg Modafinil 400 mg	Carbamazepine CR 900 mg, Pregabaline 375 mg Clomipramine SR 75 mg Modafinil 400 mg	Clomipramine SR 75 mg Oxcarbazepine 900 mg	Clomipramine SR 75 mg Oxcarbazepine 900 mg
7	Pregabaline Carbamazepine Morphium	Oxcarbazepine 900 mg, Cymbalta 60 mg 0/0/1, Tapentadol 300 mg	Oxcarbazepine 450 mg	Oxcarbazepine 900mg	Oxcarbazepine 900mg
8	-	Carbamazepine 800 mg	Carbamazepine 200 mg	Carbamazepine 200 mg	Carbamazepine 200 mg

* *taken in a context of chronic lumbovertebral pain syndrome*.

## Discussion

The CLT with MRgFUS has already been demonstrated to be a safe therapeutic option in chronic neurogenic pain with over 100 targets performed (19, 28, 29). This case series on 8 bilateral MRgFUS CLT for trigeminal pain with a mean follow-up over 4 years confirmed the very low risk profile of the intervention. It provided specific pain relief values for patients suffering from chronic therapy-resistant pain of trigeminal location. Pain relief after more than 1 year of follow-up averaged 78% (median: 75%) and was sensibly better than previously published series for other neurogenic pain locations (6, 8, 17, 19). The observed progression of pain relief over time is in accordance with a progressive reduction of the TC physiopathology (19, 24). All patients in this series acknowledged a pain relief of ≥50 % and the frequency of pain paroxysms was reduced by more than 95%. A positive bias cannot be excluded in view of the small patient number.

No lesional intervention (i.e., gamma knife surgery, radiofrequency thermocoagulation, glycerol rhizotomy, balloon compression) reached more than a low level of evidence supporting primacy over the others (34). According to our data summarized in the Introduction and to Finnerup et al. (35), trigeminal neuralgia, central post-stroke pain involving the face and central neurogenic pain associated with multiple sclerosis are recognized as neurogenic pain conditions. In this context, any further de-afferentation of the trigeminal nerve, be it either by irradiation, thermocoagulation, toxic or compression lesioning brings a risk of worsening of the neurogenic pain condition. This risk is recognized in the literature as dysesthesias or anesthesia dolorosa. As expected from the role of the medial thalamus in the TC dynamics, and as recognized in the early literature, such a iatrogenic pain production does not arise after CLT. In addition, the high plasticity of the TC network (36) can be proposed to be at the source of the absence of any somatosensory, motor or cognitive deficits, even in the acute postoperative phase: the pathophysiological basis for such a sparing capacity is the suppression of receptive fields in more than 99% of recorded CLp cells (18). These cells maintain the TC overactivity, but in addition lose their normal functions in the process, which are most probably taken over by other medial TC partners.

The CLp target, which was selected on the basis of the pathophysiological presence of low threshold calcium spike bursts discharging at 4 Hz, offers advantages over other medial thalamic targets. In contrast to the CM/Pf or to PuM, all targeted in the past (2, 7, 10, 16, 37), the CLp has known afferents from the spinothalamic tract. It is distant from primary somatosensory nuclei [ventral posterior medial (VPM) and lateral (VPL) nuclei]. An encroachment of lesioning onto adjacent structures, i.e., PuM or posterior part of MD never caused unwanted neurological or cognitive effects in the past experience (6, 8, 17, 19). The PuM provided even pain relief, which was howerver not long-lasting (16). Connections of the CLp concern large cortical domains, including areas mediating discriminative (SI, SII, posterior insula), affective-motivational (anterior cingulate, anterior insula), cognitive (prefrontal and posterior parietal cortex) and premotor aspects of pain (17, 38). This is not the case for the other medial thalamic targets.

Despite our active proposition to perform a microvascular decompression, two classical TN patients chose MRgFUS CLT and showed high pain relief at follow-up. The MRgFUS CLT represents a chance for patients who have a vascular compression but cannot or do not want to undergo a MVD.

## Conclusion

The bilateral MRgFUS CLT offers a physiopathologically based approach combining very low morbidity, good efficacy, absence of pain worsening and long term relief from neurogenic pain. Results of this small case series on chronic therapy-resistant trigeminal neuralgia, with a mean follow-up over 4 years, provides support for these characteristics in a given specific neurogenic pain location. Only a larger experience with this approach will demonstrate if it represents a treatment of first choice for patients who are not candidates for MVD.

## Data Availability Statement

The datasets generated for this study are available on request to the corresponding author.

## Ethics Statement

Ethical review and approval was not required for the study on human participants in accordance with the local legislation and institutional requirements. All patients treated with this protocol signed an informed consent form after having been fully informed about the treatment, its results and risks.

## Author Contributions

MG and DJ contributed to the conception, design of the study, acquisition, analysis, interpretation of the data and co-drafted the manuscript. DM co-drafted the manuscript. All authors read and approved the final manuscript.

## Conflict of Interest

MG, DM, and DJ were employed by SoniModul Ltd., Center of Ultrasound Functional Neurosurgery, Solothurn, Switzerland.
